# Cervical human papillomavirus: the therapeutic target of botanical drugs

**DOI:** 10.3389/fphar.2026.1822976

**Published:** 2026-04-30

**Authors:** Yan-Cai Tang, En-Feng Zhang, Han-Zhi Zhong, Mao-Ya Li, Shao-Bin Wei

**Affiliations:** 1 Gynaecology, Hospital of Chengdu University of Traditional Chinese Medicine, Chengdu, China; 2 School of Clinical Medicine, Chengdu University of Traditional Chinese Medicine, Chengdu, China

**Keywords:** botanical drugs, cervical human papillomavirus, cervicovaginal microenvironment, host-directed mechanisms, persistent infection

## Abstract

Persistent cervical human papillomavirus (HPV) infection is the key biological event underlying high-grade squamous intraepithelial lesions, cervical cancer development, and recurrence risk. Botanical drugs and their bioactive metabolites have therefore attracted interest as potential adjunctive interventions, particularly because they can modulate multiple biological pathways relevant to viral persistence and clearance. This review critically synthesizes the botanical drugs and their metabolites evaluated in cervical HPV-related studies, organizing the available evidence around experimentally interpretable therapeutic outputs. The reviewed literature indicates that specific botanical drugs are associated with the suppression of HPV oncogene expression, the disruption of viral DNA maintenance, and the modulation of critical host responses. However, the current evidence remains heterogeneous and is fundamentally limited by incomplete phytochemical characterization, variable experimental model relevance, insufficient pharmacokinetic data, and an overreliance on simplified preclinical systems. Overall, current findings support the pharmacological relevance of selected botanical drugs in persistence-related cervical HPV biology, but they do not yet establish consistent clinical efficacy. Future research should prioritize standardized botanical formulations, genotype-stratified study designs, persistence-relevant biomarkers, and durable virological and lesion-related outcomes to improve translational value.

## 1 Introduction

Cervical human papillomavirus (HPV) infection is a necessary prerequisite for cervical precancerous lesions and cancer. However, the clinically significant issue is not viral exposure itself, but the failure of viral clearance and the subsequent development of persistent infection. While most cervical HPV infections are transient, a small fraction remains detectable long-term, entering a persistent state associated with clearance difficulties, lesion progression, recurrence, and malignant transformation. This distinction is particularly crucial in cervical disease, as persistent viral activity, rather than transient positivity, is more strongly associated with high-grade squamous intraepithelial lesions (HSIL), cervical intraepithelial neoplasia (CIN) grades 2/3, and invasive cervical cancer (Molina et al., 2024). Therefore, this article specifically emphasizes the biology of cervical HPV associated with persistent infection, alongside therapeutic evidence that can be interpreted within this context.

Botanical drugs warrant pharmacological attention not merely because of their natural origins, but because they represent interventions characterized by chemical complexity and biological pleiotropy. Their activity often arises from interacting metabolites, convergent pathway modulation, and network-level effects on host tissues and the local microenvironment (Patwardhan and Gautam, 2005). Consequently, their therapeutic value cannot be deduced solely through single-target logic. Furthermore, modern botanical research must rigorously prioritize plant material authentication, the definition of medicinal materials and preparation methods, the identification of active or toxic metabolites, the selection of appropriate biological assays, and subsequent pharmacokinetic and safety evaluations. The interpretability of botanical evidence heavily relies on material characterization and model relevance. These general principles are highly applicable to cervical HPV research, as disease progression reflects continuous host-virus-microenvironment interactions rather than a simple binary infection state (Dietz et al., 2016). Against this background, this article does not treat botanical drugs as a homogeneous category with a unified mechanism. Instead, it critically evaluates each botanical drug or plant-derived metabolite, focusing on its genuine relevance to persistent cervical HPV infection, as well as the experimental interpretability and pharmacological plausibility of the associated conclusions.

Aligning with the inclusion criteria of our evidence base, this article adopts “cervical HPV infection” as the overarching disease context while strongly emphasizing “persistent infection” as the clinically meaningful focal point for evidence evaluation. By synthesizing evidence across the “target-pathway-model-result” axis, we highlight critical limitations and translational gaps. Consequently, rather than serving as a generalized overview of HPV pathogenesis, this article aims to provide a critical review of the evidence, mechanistic interpretability, and translational limitations specifically pertaining to persistent infection.

## 2 Literature search and study selection

This review applied a structured literature search and evidence selection workflow. We searched both English- and Chinese-language databases, including PubMed, Web of Science, and CNKI. Additionally, the Pharmacopoeia of the People’s Republic of China was consulted to verify official botanical nomenclature and quality control specifications. Searches combined controlled vocabulary and free-text terms (MeSH terms and keywords) using two concept blocks: (1) cervical HPV infection/human papillomavirus infection (e.g., “Human Papillomavirus Infection”, “HPV Infection”, “Cervical HPV Infection”); and (2) botanical drugs/herbal medicine and bioactive metabolites (e.g., “Botanical drugs”, “Herbal medicine”, “pharmacological effects”, together with names of specific botanical drugs/formulations and representative metabolites). The search chronology spanned from the inception of each respective database up to the final cutoff date of 28 February 2026. The complete database-specific search strings and field tags are provided in Supplementary Table S1. All records were exported to a reference manager for deduplication, and two investigators independently screened titles/abstracts and subsequently assessed full texts.

Predefined inclusion criteria: (1) Studies involving subjects or models relevant to cervical HPV infection (clinical samples, cervical/vaginal cell models), prioritizing outcomes related to persistence, clearance, recurrence, or lesion progression; (2) Botanical drug-based interventions, including single botanical drug extracts, plant-derived single entities or bioactive metabolites; (3) At least one of the following categories of outcomes was reported: virological markers; immunoinflammatory and microenvironment indicators; lesion-related cellular or histological outcomes; and evidence on mechanistic targets or signaling pathways.

For included studies, we extracted information on botanical source, compositional characterization or purity, study design, model characteristics, dosing regimen, major outcome measures, and mechanistic targets/pathways. To enhance comparability, we summarized evidence in tables aligning “model–intervention–dose–key outcomes–mechanistic targets”, and organized the synthesis across mechanistic domains relevant to HPV persistence. In this review, “botanical drugs” refer to plant-derived preparations with defined extraction/preparation processes, including extracts, enriched fractions, and their bioactive metabolites. To ensure taxonomic accuracy, all species names were validated using the Medicinal Plant Names Services (MPNS). During the literature selection process, we rigorously evaluated the chemical transparency of the interventions. Only studies describing clear extraction and preparation methods for botanical extracts were prioritized. For single or high-purity metabolites, the exact source or purity assessment methods were required. Regarding complex multi-botanical formulas or composite preparations, we prioritized studies that performed chromatographic or mass spectrometric characterization and clearly identified major metabolites. The full species names and family information, specific extraction methods, purity, and compositional characterization of the included botanical drugs are systematically summarized in Table 1.

**TABLE 1:** Mechanism-guided summary of botanical drugs and plant-derived metabolites evaluated in cervical HPV infection.

Mechanistic class	Botanical drug/preparation	Metabolites/Extracts	Preparation method	Identification method	Controls	Cells/Animals/Clinical	Model	Dose (concentration)	Duration of administration	Mechanisms	References
Disrupting HPV genome maintenance and the viral oncogene program	Coptis chinensis rhizome	Berberine	Commercially available berberine (Sigma) was freshly dissolved in DMSO [maximum 0.5% (v/v)] and then added to complete culture medium before treatment	Commercial reagent; purity >99%	Vehicle control (0.5% DMSO in media); HPV-negative cervical cancer cell line (C33a) and normal peripheral blood lymphocytes were used as controls for cytotoxicity	SiHa/HeLa	*In vitro* HPV-positive cervical cancer cell model (HPV16+ SiHa; HPV18+ HeLa)	0, 1, 10, 50, 100, 250 μg/mL	24 h for most mechanistic assays; AP-1 time-course also assessed at 0, 4, 8, 12, 18, 24 h	AP-1 activity ↓; c-Fos ↓; HPV16/18 transcription ↓; HPV E6/E7 ↓; p53 and Rb ↑; hTERT ↓; cell viability ↓; apoptosis ↑; caspase-3 activation ↑	Mahata et al. (2011)
		Berberine	Freshly prepared berberine added to culture medium	Commercial reagent; purity >99%	Untreated control; vehicle control; HPV-negative C33a and normal WRL-68 cells used in comparative assays; cisplatin positive control in selected assays	HeLa/C33a/WRL-68	*In vitro* cervical cancer cell model (HPV18+ HeLa; comparative HPV-negative C33a and normal WRL-68 cells)	150, 175, 200, 225, 250, 275, 300 μM	24 h, 48 h	cellular uptake ↑; DNA strand breaks ↑; sub-G0/G1 ↑; HPV18 E6 ↓; HPV18 E7 ↓; p53 ↑; HDAC1 ↓; HDAC2 ↓; DNA methylation-related changes; Cyclin ↓; Cdk ↓; NF-κB ↓	Saha and Khuda-Bukhsh (2014)
		Berberis aquifolium root mother tincture (BAMT)	GMP-certified commercial mother tincture; ethanol content 70% in water; diluted in complete medium before treatment	Plant-source mother tincture; reported phytochemicals analyzed by *in silico* docking; no full in-study quantitative standardization of individual constituents reported	Corresponding ethanol vehicle control; untreated control	SiHa/HeLa/C33a	*In vitro* cervical cancer cell model (HPV16+ SiHa; HPV18+ HeLa; HPV-negative C33a)	0.01%, 0.1%, 1%, 2.5%, 5%, 10% (assay-dependent)	24 h	cell viability ↓; G1 arrest ↑; STAT3 ↓; pSTAT3(Y705) ↓; JunB ↓; c-Jun ↓; HPV16/18 E6 protein ↓; HPV16/18 E7 protein ↓; predicted interference with HPV16 E6–p53/E6AP interaction	Singh et al. (2021)
	Licorice radix	Glycyrrhizin	Glycyrrhizin powder was dissolved in DMSO and added to culture medium for treatment	Commercial reagent; purity 98%	Untreated control; NAC pretreatment; caspase-8/9/3 inhibitor pretreatment controls	CaSki	*In vitro* HPV16-positive cervical cancer cell model	20, 40, 80, 160, 320 μM	24 h and 48 h for proliferation/cell death; 24 h for apoptosis, caspase, cell-cycle, qPCR, and mitochondrial assays; 12 h for ROS assay	cell viability ↓; cell death ↑; apoptosis ↑; ROS ↑; GSH ↓; mitochondrial membrane potential ↓; caspase-8/9/3 activity ↑; Bax ↑; Bad ↑; Bcl-2 ↓; G0/G1 arrest ↑; Cyclin D1 ↓; CDK4 ↓; p21Cip1 ↑; HPV16 E6 ↓; HPV16 E7 ↓; Notch1 ↓; Jagged1 ↓; Hes1 ↓	Ahmad et al. (2022)
	Sophorae flavescentis radix	Matrine	Matrine reagent was added to complete DMEM medium to the indicated treatment concentrations in the cell assays	Commercial reagent; purity >99%	Blank/untreated control; blank plasmid control in HPV E6 overexpression experime	SiHa/CaSki	*In vitro* HPV-positive cervical cancer cell model	0.25, 0.5, 1, 2, 4 mg/mL for MTT; 1 mg/mL for EdU; 0.25, 0.5, 1 mg/mL for cell-cycle assay	24, 48, 72 h for proliferation assay; 24 h for EdU, cell-cycle, and most protein/RNA assays	miR-375-3p ↑; HPV16 E6/E7 mRNA and protein ↓; Cell proliferation ↓; Apoptosis ↑; G0/G1 cell cycle arrest ↑; Cyclin D1 ↓; CDK4 ↓; p21 ↑; p27 ↑; C-myc ↓; p16 ↓; Bax ↑; Bcl-2 ↓	Zhang (2020)
​	Bruceae fructus	Bruceine D	Powder (20 mg/vial) was dissolved in DMSO and diluted with RPMI-1640 medium to prepare a 10 mmol/L stock; stored at −4 °C protected from light	HPLC purity ≥98% (supplier-reported)	Untreated control; CaSki positive control for HPV16 E6/E7 mRNA assay	Ect1/E6E7/CaSki	*In vitro* HPV16-infected immortalized cervical epithelial cell model; CaSki as HPV16-positive control	1, 5, 10, 15, 30 μmol/L	24, 48, 72 h for MTT; 48 h for cell-cycle, apoptosis and qPCR assays	cell proliferation ↓; G0/G1 arrest ↑; S phase ↓; G2/M phase ↓; apoptosis ↑; HPV16 E6 mRNA ↓; HPV16 E7 mRNA ↓	Pan et al. (2015)
			Bruceine D powder was dissolved in DMSO and diluted in RPMI-1640 medium to prepare treatment solutions	Purity >98% (supplier-reported)	Untreated control; CaSki positive control for HPV16 E6/E7 mRNA assay	Ect1/E6E7/CaSki	*In vitro* HPV16-infected immortalized cervical epithelial cell model; CaSki as positive control	1, 5, 10, 15, 30 μmol/L	24, 48, 72 h for proliferation assay; 48 h for qPCR assay	cell proliferation ↓; HPV16 E6 mRNA ↓; HPV16 E7 mRNA ↓	Sun et al. (2017)
		Brucea javanica oil emulsion (BJOE)	Commercial BJOE injection, 10 mL/ampoule, content 10%, manufactured by Shenyang Pharmaceutical University Pharmaceutical Factory	Chromatographic fingerprinting of Brucea javanica oil can be evaluated by HPLC-ELSD (Agilent Eclipse XDB-C18 column), establishing 9 common characteristic peaks for quality control and similarity evaluation	Blank control	Ect1/E6E7/CaSki	*In vitro* HPV16-positive cervical premalignant/cancer cell model	5, 10, 20, 40 μg/mL	24, 48, 72 h for MTT; 48 h for apoptosis, HPV16 E6/E7 mRNA and protein assays	cell proliferation ↓; apoptosis ↑; HPV16 E6 mRNA ↓; HPV16 E7 mRNA ↓; HPV16 E6 protein ↓; HPV16 E7 protein ↓; mutant p53 ↓; Rb ↑	Hu et al. (2013)
	Curcumae rhizoma	Zedoary turmeric oil	Provided by Hainan Bikai Pharmaceutical Co., Ltd. and incorporated into drug-containing DMEM medium at different concentrations (15, 25 and 35 μg/mL) for *in vitro* treatment	Manufacturer-supplied preparation; the article described it as a volatile oil obtained by steam distillation of Curcumae rhizoma	Untreated control	SiHa/CaSki/H8	*In vitro* HPV16-positive cervical cancer/immortalized cervical epithelial cell model	15, 25, 35 μg/mL	24 h for daily MTT readout; 5 days for cell-cycle/apoptosis and RT-PCR assays; growth curve recorded continuously for 6 days	cell viability ↓; G1 phase ↓; G2 phase ↑; S phase ↑; G2/S arrest ↑; S-phase arrest ↑; apoptosis ↑; HPV16 E6/E7 mRNA ↓	Zhang et al. (2014)
​	Scutellariae radix	Wogonin	Wogonin was isolated from the methanol extract of dried roots of *Scutellaria baicalensis* Georgi	Supplier-reported HPLC purity ≥98% (supplier-reported) Chemical structure was confirmed by HR-ESI-MS, ^1H NMR, ^13C NMR, DEPT, HMQC and HMBC.	Blank control (untreated cells); HPV-negative cervical cancer cell line (C33A) was used as a negative control to confirm E6/E7 and p53 pathway specificity;	CaSki/SiHa/C33A	*In vitro* cervical cancer cell model (HPV16+ CaSki; HPV16+ SiHa; HPV-negative C33A)	40, 80, 160 μM	48 h	cell viability ↓; apoptosis ↑; apoptotic nuclei ↑; annexin V+ cells ↑; HPV16 E6 mRNA ↓; HPV16 E7 mRNA ↓; p53 ↑; pRb ↑; phospho-pRb ↓; p21 ↑; p27 ↑; sub-G1 ↑; mitochondrial membrane potential ↓; Bax ↑; Bcl-2 ↓; cytochrome c release ↑; cleaved caspase-9 ↑; cleaved caspase-3 ↑; cleaved PARP ↑	Kim et al. (2013)
	Bupleuri radix	Aqueous extract of bupleuri radix	500 g of bupleuri radix was extracted and fractionated with petroleum ether, ether, ethyl acetate, *n*-butanol, absolute ethanol and distilled water. The aqueous fraction was collected and prepared at a concentration equivalent to 1 g/mL crude drug	NR	Untreated positive HPV-DNA amplification control	NA	*In vitro* HPV DNA assay using condyloma acuminatum isolates	0.1, 0.05, 0.025 g/mL	24 h	HPV-DNA amplification ↓	Li et al. (2005)
	Fructus akebia	α-Hederin	α-Hederin was dissolved in DMSO before cell treatment	Commercial reagent; purity ≥98%	DMSO vehicle control; DNA-damage inhibitor (MPA) control; NAC control; untreated control	SiHa/HeLa/nude mice	*In vitro* cervical cancer cell models and *in vivo* HeLa xenograft model	0, 2.5, 5, 10, 20, 40 μM for cell assays; 5 mg/kg *in vivo*	24 h for most *in vitro* assays; 48 h for clone-formation pretreatment; 21 days *in vivo*	G2/M arrest ↑; γ-H2AX ↑; p-CHK1/CHK1 ↑; P53 ↑; P21 ↑; CDC25B/CDC25C inhibition; CDK1 ↓; Cyclin B2 ↓; ATM-CHK1-CDC25B/CDC25C and ATM-P53-P21 signaling engaged; tumor growth ↓ *in vivo*	Song et al. (2025)
	Wolfiporia extensa	Pachymic acid	Pachymic acid stock solution (100 mM) was prepared in DMSO and diluted in culture medium before treatment	Commercial reagent, the purity is not reported	Untreated control; normal LO2 cells used as non-tumor comparator for proliferation assay	HeLa/SiHa/LO2	*In vitro* cervical cancer cell models; HeLa used for TEM and Western blot validation	0, 10, 20, 30 μM	24 h for most mechanistic assays; proliferation also assessed at 12, 24, 48, and 72 h	LC3-II/LC3-I ↑; p-AKT ↓; p-mTOR ↓; HDAC6/HK2/PIP4K2C/RAB7A/HMOX1/VPS4B implicated as autophagy-related target genes; autophagy induction via AKT/mTOR inhibition	Wu (2021)
Reprogramming mucosal immunity and weakening immune evasion nodes	Coptis chinensis rhizome	Berberis aquifolium root mother tincture (BAMT)	GMP-certified commercial mother tincture; ethanol content 70% in water; diluted in complete medium before treatment	Plant-source mother tincture; reported phytochemicals analyzed by *in silico* docking; no full in-study quantitative standardization of individual constituents reported	Corresponding ethanol vehicle control; untreated control	SiHa/HeLa/C33a	*In vitro* cervical cancer cell model (HPV16+ SiHa; HPV18+ HeLa; HPV-negative C33a)	0.01%, 0.1%, 1%, 2.5%, 5%, 10% (assay-dependent)	24 h	STAT3 ↓; pSTAT3(Y705) ↓; JunB ↓; c-Jun ↓; immune-evasion-associated transcriptional signaling ↓; HPV E6/E7 ↓	Singh et al. (2021)
	Borneol	Borneol	Commercially sourced Borneol (Aladdin, B119291) was dissolved to a working concentration of 100 μg/mL	NR	PBS vehicle control; Blank control (untreated cells or mice)	Macrophages (Raw 264.7), Dendritic cells (DC 2.4), HPV E6/E7-expressing mouse lung epithelial cells (TC-1), and mouse splenic lymphocytes; Female C57BL/6 mice	*In vitro* macrophage inflammatory activation model; DC inflammatory activation model; TC-1/splenic lymphocyte transwell coculture model; *in vivo* LPS/TC-1 cervical orthotopic injection model	100 μg/mL	24 h for most cell experiments; 1 day for TC-1 E6/E7 mRNA readout; 3 days for TC-1 viability; 14 days endpoint in mouse model after repeated lavage treatment	IL-6 ↓; IL-1β ↓; TLR4 ↓; TLR9 ↓; p-p65 ↓; p-p65 nuclear translocation ↓; iNOS ↓; IL-10 ↑; TGF-β ↑; TNF-α ↑; CASP1 ↑; IL-12 ↑; IL-23 ↑; HPV E7 mRNA ↓; CD274/PD-L1 mRNA ↓; p-STAT3 ↓; CD274/PD-L1 protein ↓; CD3^+^CD8^+^ T cells ↑; CD8+/CD4+ ratio ↑; TC-1 cell viability ↓; HPV E6 mRNA ↓; HPV E7 mRNA ↓	Liu et al. (2025)
	Baofukang suppository	Zedoary turmeric oil and borneol	Commercial suppository from Hainan Bikai Pharmaceutical Co., Ltd. (approval no. Z46020058)	Manufacturer-defined pharmaceutical preparation, 1.74 g per suppository; no additional in-study physicochemical characterization reported	Interferon suppository control	Clinical patients with cervical HPV infection	Randomized controlled clinical study	1 suppository/day	3 months	clinical efficacy ↑; HPV negative-conversion rate ↑; CD3^+^ ↑; CD4^+^ ↑; CD4+/CD8+ ↑; CD8^+^ ↓; IL-2 ↓; IL-1β ↓; pyorrhea score ↓; abnormal leucorrhea score ↓; cervical erosion score ↓	Tian et al. (2024)
					Interferon group; Baofukang + interferon combination group	Clinical patients with cervical HPV infection	Randomized controlled clinical study	1 suppository/day	3 months	Th1-cell proportion ↑; T-bet mRNA ↑; Th2-cell proportion ↓; GATA-3 mRNA ↓; LCT normalization ↑	Zhao et al. (2023)
Resolving chronic inflammation and inflammatory tolerance niches	Sophorae flavescentis radix	Matrine	Purified matrine dissolved for cell and animal treatment	Commercial reagent; HPLC purity ≥98%	Untreated control; pc-NC control; HMGB1 overexpression rescue	HeLa/nude mice	*In vitro* HPV-positive cervical cancer cell model and HeLa xenograft model	5 mmol/L *in vitro*; 50 mg/kg *in vivo*	24 h *in vitro*; every other day for 7 injections *in vivo*.	cell viability ↓; apoptosis ↑; invasion ↓; migration ↓; vascular mimicry ↓; tumor growth ↓; CD31-positive angiogenesis ↓; HMGB1 ↓; RAGE ↓; HMGB1 overexpression partially reverses antitumor effects	Ran et al. (2026)
	Isodon japonicus	Glaucocalyxin B	Prepared a 10 mM stock solution, 3.7 mg of Glaucocalyxin B powder was dissolved in 1 mL of DMSO. The resulting solution was subsequently aliquoted and stored at −20 °C until further use	Commercial reagent; HPLC purity ≥98%	Untreated control; NAC pretreatment control	C33A/HeLa/SiHa	*In vitro* cervical cancer cell models (HPV-negative C33A; HPV18-positive HeLa; HPV16-positive SiHa)	C33A: 0–20 μM for viability/ROS; 10 μM for apoptosis/autophagy; HeLa and SiHa: 0–20 μM for ROS; 40 μM for apoptosis/autophagy; NAC 5 mM pretreatment	Mainly 24 h; clonogenic assay 3 days	ROS ↑; cell viability ↓; clonogenic growth ↓; cleaved PARP ↑; LC3 ↑; NAC pretreatment partially reverses growth inhibition and reduces cleaved PARP/LC3, supporting ROS-dependent apoptosis and autophagy	Liang L (2022)
	Baofukang suppository	Zedoary turmeric oil and borneol	Commercial suppository from Hainan Bikai Pharmaceutical Co., Ltd. (approval no. Z46020058)	Manufacturer-defined pharmaceutical preparation, 1.74 g per suppository; no additional in-study physicochemical characterization reported	Recombinant human interferon α-2b gel alone	Clinical patients with cervical HPV infection	Randomized controlled clinical study	1 suppository, every other day	12 weeks	cervical inflammation score ↓; TNF-α ↓; TGF-β1 ↓	Song and Bai (2024)
Restoring cervicovaginal homeostasis	Sophora flavescens gel	Total alkaloids of sophora	Commercial gel preparation (5 g/piece), produced by Guiyang Xintian Pharmaceutical Co., Ltd. (GYZZ Z20050058)	The total alkaloid content of sophorae flavescentis radix is 100 mg per sachet.	Recombinant human interferon α-2b gel control; combination group (Sophora flavescens gel + interferon α-2b gel)	Clinical patients with cervical HPV infection	Randomized controlled clinical study	1 g/day	3 months	HPV viral load ↓; cure rate ↑; total effective rate ↑; HPV16 viral load/response ↑; HPV18 viral load/response ↑	Zhao et al. (2016)
					Blank control/follow-up only	Clinical patients with cervical HPV infection	Prospective randomized controlled clinical study	5 g/day	3 months (with clinical follow-ups at 3 and 6 months)	HPV clearance ↑; total effective rate ↑; mixed HPV infection response ↑; INF ↓; vaginal inflammatory reaction ↓; vaginal microecology ↑	Liang (2018)
	Bruceae fructus	Brucea javanica seed infusion	Under sterile conditions, dried Brucea javanica seeds were peeled, kernels were crushed, soaked in 95% ethanol for 1 week, filtered, and used after near-complete evaporation of ethanol; topically applied to the cervix by cotton swab	In-house hospital preparation; no additional physicochemical standardization reported	Blank untreated control	Clinical patients with cervical HPV infection	Randomized controlled clinical study	Topical cervical application once daily	7 days per course, 3 courses; 2-week interval between courses	high-risk HPV viral load ↓/HPV response rate ↑; CINI regression ↑; local tolerability acceptable	Huang (2016)
	Baofukang gel	Zedoary turmeric oil and borneol	Commercial gel preparation (4 g/tube) (approval no. Z20050474)	Manufacturer-defined pharmaceutical preparation; no additional in-study physicochemical characterization reported	No-treatment control	Clinical patients with cervical HPV infection	Randomized controlled clinical study	1 application/day	10 days per course; 3 courses	HPV negative-conversion ↑; HPV titer ↓; clinical tolerability acceptable	Han (2014)
					No-treatment control	Clinical patients with cervical HPV infection	Randomized controlled clinical study	1 application/day	10 days per course; 3 courses	HPV negative-conversion ↑; cervical columnar ectopy area ↓; local epithelial improvement ↑	Zhang et al. (2013)
					No-treatment control	Clinical patients with cervical HPV infection	Randomized controlled clinical study	1 application/day	10 days per course; 3 courses	high-risk HPV negative-conversion ↑; local tolerability acceptable	Liu (2014)
​	Baofukang suppository	Zedoary turmeric oil and borneol	Commercial suppository from Hainan Bikai Pharmaceutical Co., Ltd. (approval no. Z46020058)	Manufacturer-defined pharmaceutical preparation, 1.74 g per suppository; no additional in-study physicochemical characterization reported	Recombinant human interferon α-2b gel alone	Clinical patients with cervical HPV infection	Randomized controlled clinical study	1 suppository, every other day	12 weeks	vaginal cleanliness grade ↓; Nugent score ↓; HR-HPV RLU/CO ↓; cervical inflammation score ↓; clinical response rate ↑	Song and Bai (2024)
					α-2b interferon suppository control	Clinical patients with cervical HPV infection	Randomized controlled clinical study	1 suppository/day	3 months	overall response rate ↑; HPV negative-conversion/clearance ↑; vaginal pH ↓; Nugent score ↓; adverse reactions ↓	Liu (2024)

Data are summarized from *in vitro*, *in vivo*, and clinical studies evaluating botanical drugs, extracts, and their active constituents in models of cervical HPV infection. The botanical drug names in the table were standardized according to the Medicinal Plant Names Services (MPNS).

↑ and ↓ indicate increases or decreases relative to the corresponding control or model groups; NR, not reported; HPV, human papillomavirus; DMSO, dimethyl sulfoxide; ELNs, exosome-like nanoparticles; PBS, phosphate-buffered saline; BMDCs, bone marrow-derived dendritic cells; MoDCs, monocyte-derived dendritic cells; HPLC, high-performance liquid chromatography; LPS, lipopolysaccharide; IFA, incomplete Freund’s adjuvant.

To provide a basic assessment of study quality, we applied a simplified, reporting-based checklist of key bias-sensitive items to each included study. This evaluation focused on three primary domains: (1) the clarity of control conditions, including negative controls and matched vehicle or solvent controls; (2) the comprehensiveness of exposure details, such as dose or final concentration, route, frequency, timing, and duration; and (3) the adequate characterization of the botanical drug interventions, encompassing extract preparation, chemical profiling, and metabolite source and purity. Reporting completeness was coded as R, P, or NR to reflect its potential impact on interpretability. Crucially, this coding system was employed as an objective reporting checklist rather than a subjective risk-of-bias rating. The results are detailed in Supplementary Table S2, which provides a study-by-study snapshot of reporting completeness across these essential items.

## 3 Pathogenesis of cervical HPV infection

The development of persistent cervical HPV infection is fundamentally the process by which a virus, typically controlled or cleared at the mucosal surface, successfully establishes a long-term epithelial reservoir. HPV enters basal cells through mucosal micro-abrasions, maintains its genome within infected keratinocytes, and couples its life cycle to the epithelial differentiation program, thereby achieving persistence under conditions of limited early immune recognition (Ntuli et al., 2022). While this infection is ultimately cleared in most women, when viral genome maintenance outlasts the host’s effective immune control, the infected epithelium is gradually remodeled into a state highly conducive to the survival of HPV-positive cells (Guo et al., 2023). In the cervix, this persistence-prone state is clinically significant because it drives the transition from a merely detectable infection to a condition characterized by clearance difficulties, recurrent lesions, high-grade squamous intraepithelial lesions, and ultimately, invasive malignancy (Xu et al., 2025).

This transition is driven not merely by viral presence *per se*, but rather by dynamic interactions among early viral gene activity, host DNA damage and repair responses, epithelial turnover, local immune control, and the cervicovaginal tissue microenvironment. Persistent E6/E7 activity attenuates cell cycle checkpoint regulation and sustains the proliferation of infected cells (Xu and Tong, 2025). Concurrently, replication stress and oxidative DNA damage exacerbate genomic instability, establishing the requisite conditions for viral integration, an event intimately linked to malignant progression. At the tissue level, HPV prolongs its residency by restricting effective antiviral sensing and delaying immune-mediated clearance. Simultaneously, alterations in the local microbiome architecture, barrier integrity, inflammatory signaling, and intercellular crosstalk collectively reinforce a local cervical milieu that opposes natural regression, favoring instead persistence, recurrence, and lesion evolution (Zhuoyao et al., 2025; Cui et al., 2025). Consequently, persistent infection should not be conceptualized as a mere virological endpoint, but rather as a dynamic state encompassing the host, virus, and microenvironment, yielding measurable virological, epithelial, immunological, and pathological consequences.

Building upon this foundation, subsequent subsections will dissect persistent cervical HPV infection by focusing on specific pathogenic nodes that are both experimentally interpretable and highly relevant to pharmacological intervention and evidence evaluation. This structural approach ensures that the ensuing analysis of botanical evidence remains rigorously anchored to the biology underlying persistent infection.

### 3.1 Viral oncogene program and checkpoint collapse

A defining feature of hrHPV persistence is sustained activity of the early gene program, which couples viral genome maintenance to host cell-cycle reprogramming. In infected epithelia, replication stress and DNA lesions engage the DNA damage response (DDR)—a checkpoint and repair signaling network activated by stalled replication forks and genotoxic stresses, including viral infection (Scully et al., 2019; Weitzman and Fradet-Turcotte, 2018). hrHPV can modulate DDR signaling to create a replication-permissive cellular state that supports viral genome replication and maintenance; however, chronic rewiring of these processes is also associated with increased DNA breaks, chromosomal instability, and a higher propensity for lesion progression (Groelly et al., 2023; Studstill and Moody, 2023; Sriramana et al., 2022; Jones et al., 2024; Goldstein et al., 2023; Naderzadeh et al., 2025; Schott et al., 2024; Zech et al., 2024). A critical driver of these DNA lesions is oxidative stress. Elevated Reactive Oxygen Species (ROS) directly oxidize nucleoside bases, such as forming 8-oxoguanine. When attempted Base Excision Repair (BER) of these oxidized bases occurs simultaneously on opposing DNA strands, it inadvertently generates DNA double-strand breaks (DSBs). Furthermore, ROS-induced oxidized bases act as physical obstacles to the replication machinery, causing replication fork breakdown and yielding additional DSBs. Crucially, the accumulation of these ROS-induced DSBs provides the necessary physical substrates—open DNA break ends—that facilitate the genomic integration of high-risk HPV, thereby cementing persistent infection and driving malignant transformation (Sai Srinivasa et al., 2019). At the core of this persistence-favoring state is checkpoint collapse driven by the viral oncogenes E6 and E7. E6 binds the intracellular ubiquitin ligase E6-associated protein (E6-AP) to form a complex that promotes proteasome-dependent degradation of p53, thereby weakening p53-mediated growth restraint and damage-induced checkpoint control. In parallel, E7 targets the growth inhibitory protein pRb, functionally inactivates it, and releases the transcription factor E2F, which drives unscheduled S-phase entry, abnormal proliferation, and malignant transformation (Guo et al., 2011). Together, loss of p53–pRb control uncouples cell-cycle progression from checkpoint surveillance, amplifies replicative stress, and promotes genomic instability—mechanistic features that directly link persistent viral oncogene output to long-term maintenance of infected cells and to progression of cervical lesions.

### 3.2 Immune evasion and mucosal immune tolerance

HPV persistence is strongly supported by immune evasion strategies that collectively blunt antiviral sensing, dampen interferon output, and prevent the formation of effective antigen-specific clearance. At the innate immune level, high-risk HPV early proteins can reduce type I interferon production through multiple mechanisms, including upregulation of the H3K9 methyltransferase SUV39H1 and blockade of the cGAS–STING–TBK1 axis, thereby limiting host recognition of HPV DNA and weakening early antiviral responses (Lo Cigno et al., 2020b). HPV can also impair RNA-sensing pathways: early proteins bind upstream regulators (TRIM25 and USP15) of the cytoplasmic RNA sensor RIG-I, promote TRIM25 ubiquitination/degradation, inhibit RIG-I interaction with MAVS, and consequently attenuate antiviral signaling (Chiang et al., 2018). At the adaptive immune level, persistence is further enabled when antigen processing/presentation and downstream T-cell priming/effector activity are functionally constrained, allowing infected epithelial cells to remain “immunologically silent” and extend the window for viral maintenance. Importantly, immune escape in the cervix is not a single-cell event but a tissue-level phenomenon shaped by immune-responsive neighboring cells—including neutrophils, dendritic cells, macrophages, T cells, natural killer cells, Langerhans cells, and stromal fibroblasts—which together determine the local immune landscape and tolerance-prone microenvironment (Cigno et al., 2024; Junjun et al., 2022; Mohammed et al., 2025; Yang and Xue, 2024; Premkumar et al., 2022). This immune context provides a mechanistic bridge to therapeutic strategies aimed at restoring interferon-linked antiviral tone, improving antigen presentation and T-cell engagement, and remodeling tolerance-associated immune crosstalk.

### 3.3 Chronic inflammatory and oxidative remodeling

Inflammation fundamentally serves as a protective response against harmful irritants, damaged cells, or invading pathogens such as HPV (Moody, 2022). However, during cervical HPV infection, this inflammatory signaling is pathologically re-tuned. Infected or stressed epithelial cells, alongside the surrounding stromal and immune compartments, generate an array of cytokines, chemokines, reactive oxygen species (ROS), and nitric oxide, collectively reshaping immune-cell recruitment, effector functions, and tissue repair programs. Crucially, this microenvironmental remodeling is profoundly orchestrated by danger-associated molecular pattern (DAMP) signals, which couple epithelial stress to self-amplifying inflammatory loops. As a central nexus between stress sensing and inflammatory amplification, activation of the HMGB1-RAGE axis enhances NF-κB-dependent transcriptional programs, sustaining the production of pro-inflammatory mediators and ROS (Xu et al., 2012). This establishes a pathological milieu characterized by prolonged damage signaling and delayed inflammatory resolution. Consequently, a reprogrammed balance of inflammatory mediators emerges, supporting the viral life cycle and fostering a permissive state where chronic inflammatory activation coexists with incomplete antiviral clearance (Shamseddine et al., 2021; Lo Cigno et al., 2020a). In this setting, immune pressure is insufficient for viral eradication but adequate to drive ongoing tissue remodeling. Ultimately, HPV leverages this chronic inflammatory tone not only to sustain persistence but also to facilitate carcinogenic processes. Therefore, inflammatory and oxidative remodeling in cervical HPV disease should not be viewed merely as the passive release of local mediators, but rather as the systemic output of host danger-sensing circuits—a central mechanistic link dictating whether inflammation is resolved or redirected toward pro-tumorigenic tissue reprogramming (Bruno et al., 2025; Gachpazan et al., 2025; Marcos et al., 2024; Oliveri et al., 2025; Ovestad et al., 2024).

### 3.4 Microbial imbalance and barrier dysfunction

Imbalance in the lower genital tract microbiota contributes to HPV persistence and progression to precancerous lesions and cancer (Wei et al., 2022; López-Filloy et al., 2022; Kombe Kombe et al., 2020). One mechanistic entry point is barrier integrity: disruption of mucosal protective factors can weaken epithelial barrier functions, increasing susceptibility to HPV invasion, persistent infection, and genomic integration (Yang et al., 2023). Conversely, HPV infection may also perturb the genital microbiota, supporting a bidirectional virus–microbiota interaction that can influence HPV clearance and persistence (Kyrgiou et al., 2017; Zhou and Ding, 2019). Within a dysbiotic state, local biochemical conditions can shift in ways that favor pathogen colonization and microenvironmental instability. When microecological balance is disrupted in the setting of HPV infection, redox reactions and intracellular accumulation of H_2_O_2_ may occur, accompanied by the generation of alkaline components; these changes can counter local acidification and create conditions permissive for pathogenic overgrowth (Huang et al., 2024; Liu et al., 2023). In parallel, metabolites produced by vaginosis-associated and pathogenic bacteria—such as glucuronidase, acetylglucosaminidase, and sialidase—can remodel glycosylation states of membrane glycoproteins, thereby altering membrane protein function, disturbing cellular signaling and endocytic processes, and ultimately heightening susceptibility to HPV infection and its persistence (Liu et al., 2021; Arash et al., 2019). Collectively, dysbiosis and HPV infection may reinforce each other, sustaining barrier impairment and inflammatory tone and promoting HPV-related disease progression.

These mechanisms do not operate in isolation; instead, they form a self-reinforcing loop that stabilizes hrHPV persistence and promotes lesion evolution. At the center, the viral oncogene program (E6/E7) collapses p53/pRb checkpoints and sustains replicative stress, creating a permissive cellular state for long-term viral maintenance and progressive genomic instability. This state is prolonged by immune evasion, in which innate nucleic-acid sensing and interferon-linked antiviral tone are dampened and adaptive immune clearance is functionally constrained. Persistent infection then sustains a chronic inflammatory and oxidative milieu that remodels epithelial and stromal programs, while barrier impairment and inflammation are tightly coupled to cervicovaginal dysbiosis. Dysbiosis-associated redox and enzymatic shifts can further amplify inflammatory tone and epithelial susceptibility, reinforcing the persistence-prone microenvironment. Together, checkpoint collapse–immune escape–inflammation/oxidative remodeling–dysbiosis constitute an interconnected pathogenic circuit that extends the persistence window and accelerates the transition from infection to high-grade lesions. Within this circuit, ROS acts as a critical mechanistic bridge, translating chronic mucosal inflammation into the DNA double-strand breaks strictly required for viral genomic integration.

**FIGURE 1:**
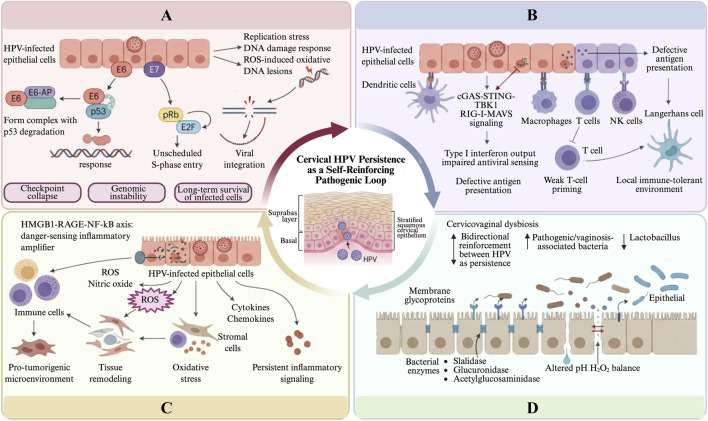
Pathogenesis of Cervical HPV Infection: A Self-Reinforcing Pathogenic Loop. **(A)** Viral oncogene program and checkpoint collapse: persistent HPV E6/E7 activity disrupts p53/pRb checkpoint control and sustains aberrant proliferation, while replication stress and DNA damage promote genomic instability and viral maintenance. **(B)** Immune evasion and mucosal immune tolerance: HPV persistence is reinforced by suppression of antiviral sensing (e.g., cGAS-STING, RIG-I) and impaired antigen presentation, leading to a tolerance-prone mucosal immune environment. **(C)** Chronic inflammatory and oxidative remodeling: chronic activation of HMGB1-RAGE-NF-κB signaling and ROS/cytokine networks drives inflammatory and oxidative remodeling without effective viral clearance. **(D)** Microbial imbalance and barrier dysfunction: cervicovaginal dysbiosis and barrier dysfunction reduce mucosal defense, alter local biochemical conditions, and increase epithelial susceptibility to persistent HPV infection.Abbreviations: p53, Tumor Protein p53; pRb, Retinoblastoma Protein; E2F, Early 2 Transcription Factor; ROS, Reactive Oxygen Species; cGAS, cyclic GMP-AMP synthase; STING, stimulator of interferon genes; TBK1, TANK Binding Kinase 1; RIG-1, Retinoic Acid-Inducible Gene 1; MAVS: Mitochondrial Antiviral Signaling Protein; HMGB1, High Mobility Group Box-1 Protein; RAGE, Receptor for Advanced Glycation Endproducts; NF-κB, Nuclear Factor kappa-light-chain-enhancer of activated B cell.

The pathogenesis of persistent cervical HPV infection is summarized in Figure 1.

## 4 Therapeutic mechanisms of botanical drugs against cervical HPV infection

Botanical drugs and plant-derived metabolites have been reported to influence cervical HPV infection through multiple, experimentally trackable modes of action. Across HPV-relevant systems, representative effects include reducing viral oncogene output and destabilizing viral DNA states that support persistence, reshaping mucosal immune activation and antigen-specific responses, attenuating inflammation/oxidative signaling that sustains a tolerance-prone milieu, and improving cervicovaginal ecological conditions linked to barrier integrity and pathogen burden. These mechanistic actions are typically accompanied by verifiable downstream readouts, such as restoration of cell-cycle control, apoptosis/autophagy outputs, improvement of local inflammatory profiles, and HPV typing/clearance outcomes. To ensure mechanistic precision, this review conceptually distinguishes between direct antiviral effects and host-directed antitumor or cytotoxic effects. Direct antiviral effects refer to actions on HPV-related molecular endpoints and on upstream transcriptional regulators linked to viral oncogene expression. Conversely, host-directed antitumor or cytotoxic effects refer to downstream cellular consequences in HPV-positive cells, such as the restoration of checkpoint control, inhibition of proliferation, apoptosis, or other lesion-related cellular responses. Representative direct antiviral readouts include reduction of viral oncogene output and destabilization of viral DNA states that support persistence, whereas downstream outcomes such as apoptosis, restoration of cell-cycle control, and inhibition of proliferation are interpreted as consequential cellular responses rather than standalone evidence of direct antiviral action (Simran et al., 2021; Dreer et al., 2016).

When evaluating these host-directed botanical interventions, it is crucial to delineate the genotype specificity of the current evidence. Preclinical mechanistic studies, particularly those evaluating targeted active metabolites, have predominantly relied on HPV16- and HPV18-positive models. The current evidence for these specific agents is largely genotype-specific, with most studies demonstrating targeted suppression of HPV16/18 E6/E7 oncoproteins and related promoter activities. Conversely, the evidence supporting complex botanical formulations suggests a broad-spectrum therapeutic potential. Because the primary pharmacological actions of these complex formulations are grounded in host microenvironmental reconditioning, their efficacy relies on correcting shared oncogenic niches rather than targeting a single viral genotype’s sequence. Therefore, while targeted botanical metabolites currently present genotype-specific evidence, immunomodulatory and microecological botanical formulations offer a broad-spectrum approach to high-risk HPV clearance.

**FIGURE 2:**
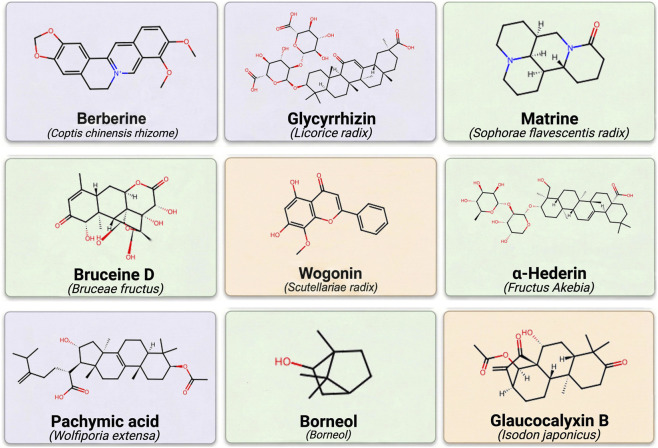
Representative Plant-Derived Metabolites from Botanical Drugs Relevant to Cervical HPV Infection (Representative small-molecule metabolites discussed in this review. Chemical structures were refernced from the PubChem database, and drawn using ChemDraw).

**FIGURE 3:**
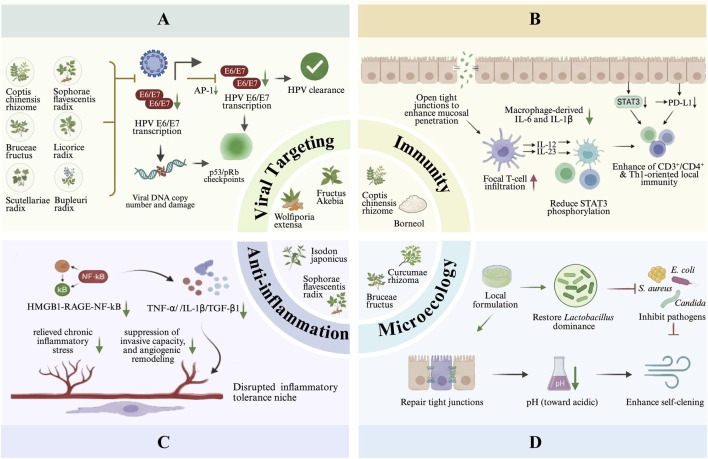
Therapeutic Mechanisms of Botanical Drugs Against Cervical HPV Infection. **(A)** Disrupting HPV genome maintenance and oncogene program: botanical drugs or bioactives reduce HPV DNA burden and suppress E6/E7 (e.g., via AP-1 inhibition or miRNA-mediated regulation), restoring p53/pRb checkpoints and promoting viral clearance. **(B)** Reprogramming mucosal immunity and weakening immune evasion: by improving local penetration, attenuating STAT3/PD-L1-mediated immune evasion, and enhancing IL-12/IL-23-associated T-cell responses. **(C)** Resolving chronic inflammation and oxidative stress: anti-inflammatory actions dampen HMGB1-RAGE-NF-KB signaling and reduce cytokine/ROS-driven stress, thereby restraining inflammation-associated tissue remodeling. **(D)** Restoring cervicovaginal homeostasis and microbiota balance: therapies restore *Lactobacillus* dominance, suppress pathogens, repair epithelial tight junctions and normalize vaginal pH, thereby improving mucosal barrier function and self-clearing capacity. Abbreviations: p53, Tumor Protein p53; pRb, Retinoblastoma Protein; IL-6/12/23/1ẞ, interleukin-6/12/23/1ẞ; STAT3, Signal Transducer and Activator of Transcription 3; PD-LI, Programmed Cell Death Ligand 1; HMGB1, High Mobility Group Box-1 Protein; RAGE, Receptor for Advanced Glycation Endproducts; NF-kB, Nuclear Factor kappa-light-chain-enhancer of activated B cell; TNF-α, Tumor Necrosis Factor-a; TGF-B1, Transforming Growth Factor-ẞ1; *E. coli*, *Escherichia coli*; *S. aureus*, *Staphylococcus aureus*.

Using this mechanistic-evidence framework, Sections 4.1–4.4 synthesize available data by grouping studies according to their dominant action patterns and measurable endpoints. Table 1 summarizes the botanical drugs and active metabolites discussed. Figure 2 presents only representative metabolites substantively discussed in the main text and retained after evidence-tier review. The integrated schematic of proposed mechanisms is presented in Figure 3.

### 4.1 Disrupting HPV genome maintenance and the viral oncogene program

Plant-derived metabolites have been reported to curb persistence by directly reducing viral oncogene output and/or destabilizing viral DNA states that support maintenance. Accordingly, this section explicitly organizes evidence around measurable endpoints tightly linked to viral control, including E6/E7 transcripts or proteins, upstream transcriptional readouts such as AP-1 activity, and viral DNA copy-number or damage/persistence-related readouts. This restructuring helps distinguish direct interference with HPV-maintenance programs from broader cytotoxic effects observed in HPV-positive cancer cells, while still acknowledging the accompanying downstream cellular consequences (Simran et al., 2021; Dreer et al., 2016).

#### 4.1.1 Suppressing E6/E7 and upstream transcriptional regulators

Persistent expression of HPV E6 and E7 oncogenes constitutes the central molecular driver of HPV-associated lesion maintenance. These viral proteins destabilize host tumor suppressor pathways and sustain proliferative signaling. Therefore, direct suppression of E6/E7 expression or interference with their upstream transcriptional regulators represents a plausible mechanistic entry point for preclinical intervention.

The predominant active metabolite of Coptis chinensis rhizome (Wang et al., 2023) (*Coptis chinensis* Franch.; Ranunculaceae) is berberine, a natural isoquinoline alkaloid characterized by extensive pharmacological validation and a well-documented safety profile (Zhou et al., 2020). Berberine has been shown to suppress the transcriptional activity of activator protein-1 (AP-1), a host factor essential for regulating HPV oncogene expression, thereby reducing the levels of viral oncoproteins E6 and E7 (Mahata et al., 2011). Crucially, evidence indicates that this suppression is not an indiscriminate blockade of physiological AP-1 signaling. Rather, berberine selectively targets the constitutively active AP-1 complexes required to sustain transcription from the viral upstream regulatory region (URR). Mechanistically, berberine alters AP-1 composition by downregulating c-Fos—a key oncogenic component enriched in HPV-transformed cells—while sparing other transcription factors like Sp1 and preserving normal epithelial cell viability. Consequently, as an upstream transcriptional node, AP-1 represents a primary mechanistic entry point through which berberine dismantles HPV-driven oncogenic programs. Further corroboration for this transcriptional suppression emerges from studies on berberine-containing botanical preparations. In HPV16-positive SiHa and HPV18-positive HeLa cells, the berberis aquifolium root mother tincture (BAMT) reduced cell viability and induced a modest G1-phase arrest. This treatment specifically decreased the AP-1 components JunB and c-Jun, precipitating a gradual loss of HPV E6 and E7 proteins. Beyond transcriptional control, the same study utilized molecular docking to propose that berberine physically disrupts the interaction of HPV16 E6 with p53, E6AP, or both. This suggests a dual mechanism where berberine not only inhibits viral oncogene transcription but also directly destabilizes the resulting oncoprotein complexes (Singh et al., 2021). Extending beyond targeted transcription-factor modulation, mechanistic investigations in HeLa cells indicate that berberine disrupts the HPV oncogene program via broader epigenetic and nuclear regulatory processes. Following cellular uptake, berberine treatment triggered the accumulation of DNA strand breaks, altered methylation profiles, and reduced HDAC1/HDAC2 expression, concurrently downregulating HPV18 E6/E7 (Saha and Khuda-Bukhsh, 2014). These observations imply that the suppression of viral oncogene output is intricately linked to extensive transcriptional and epigenetic remodeling within HPV-positive cervical cancer cells. Collectively, these findings delineate a multi-layered model: berberine attenuates upstream transcriptional regulators of HPV oncogenes while simultaneously reshaping the nuclear landscape required for their maintenance. However, because these insights derive primarily from *in vitro* tumor models, they provide a mechanistic rationale for oncogene destabilization rather than definitive clinical proof of clearance in persistent *in vivo* cervical HPV infections.

Glycyrrhizin is the major bioactive of licorice radix (Peraza-Labrador et al., 2022) (*Glycyrrhiza uralensis* Fisch. ex DC.; Fabaceae), with well-recognized anti-inflammatory and antiviral activities. In HPV16-positive CaSki cervical cancer cells, glycyrrhizin directly suppresses the expression of the viral oncogenes E6 and E7, placing it within the group of botanical metabolites that act on the oncogene-supportive core of HPV-driven malignancy. Mechanistically, this effect is accompanied by inhibition of the Notch signaling pathway, including reduced expression of Notch1, Jagged1, and Hes1, suggesting that glycyrrhizin does not merely lower viral oncogene output in isolation, but disrupts a broader host-supported regulatory context permissive for maintenance of the transformed state. In this framework, E6/E7 suppression and Notch attenuation should be interpreted as parallel interference with interconnected pro-survival and pro-proliferative programs, rather than as nonspecific secondary effects of generalized cytotoxicity. Because persistent E6/E7 expression is required to sustain HPV-dependent malignant phenotypes, and Notch-associated signaling can reinforce proliferative and cell-fate programs in cervical cancer cells, this pattern supports the view that glycyrrhizin weakens HPV-driven oncogene maintenance at the level of both viral output and host regulatory circuitry.

The principal active metabolites of Sophorae flavescentis radix (Zhang et al., 2023) (*Sophora flavescens* Aiton; Fabaceae) comprise the alkaloids matrine and oxymatrine, alongside sophora flavanone glycosides and related metabolites (Ma et al., 2023). Matrine has been reported to suppress HPV E6/E7 transcription. Mechanistically, matrine markedly elevates miRNA-375-3p levels, which in turn suppresses HPV16 E6/E7 transcription (Zhang, 2020). These data support a pathway in which matrine engages a miRNA regulatory node and produces a measurable reduction in viral oncogene output, positioning E6/E7 repression as the proximate antiviral endpoint. Future work should clarify whether this regulatory axis is preserved in organotypic cervical epithelial models and *in vivo* and define exposure–response relationships compatible with clinical dosing.

Bruceae fructus (Man and Choo, 2017) (*Brucea javanica* (L.) Merr.; Simaroubaceae) has been reported to reduce HPV16 E6 and E7 mRNA expression as proximate antiviral evidence, which is accompanied by downstream antitumor effects (Fan et al., 2022), including induction of apoptosis and time- and concentration-dependent inhibition of Ect1/E6E7 cells *in vitro* (Pan et al., 2015; Sun et al., 2017). In parallel, Brucea javanica oil emulsion (BJOE) decreases HPV16 E6/E7 protein levels, driving the downstream suppression of proliferation in HPV16-infected cells *in vitro* (Guo et al., 2012; Hu et al., 2013). Together, these preparations link Bruceae fructus to measurable reductions in viral oncogene output, although the chemical complexity of BJOE and incomplete attribution of activity beyond a limited number of metabolites remain major barriers to reproducible pharmacology and translation.

Curcumae rhizoma (*Curcuma phaeocaulis* Valeton; Zingiberaceae) is widely cultivated in tropical south and southwest Asia. Zedoary turmeric oil (Li et al., 2018), a volatile oil obtained by steam distillation of curcumae rhizoma, has demonstrated significant anti-cancer properties. An experimental investigation was conducted in which HPV16-positive cervical cancer cell lines (SiHa, CaSki) and cervical immortalized cell lines (H8) were cultured in media containing varying concentrations of zedoary turmeric oil. Data revealed that zedoary turmeric oil exerts an inhibitory effect on cell proliferation across a spectrum of concentrations. The incidence of apoptosis in SiHa, CaSki, and H8 cell lines within the treatment group was significantly higher than that in the control. Furthermore, the transcriptional levels of the HPV16 E6/E7 genes were substantially diminished in these three cell lines in comparison with the control (Zhang et al., 2014). Considering the complex composition of Curcumae rhizoma, an examination of the specific roles of its monomeric metabolites, including B-elemene and curcumol, may provide valuable insights for the development of clinical approaches and pharmaceutical formulations targeting HPV infection (Xin, 2013).

Scutellariae radix (Zhou et al., 2023) (*Scutellaria baicalensis* Georgi; Lamiaceae) is a well-known source of bioactive metabolites. Wogonin, an O-methylflavonoid extracted from this botanical drug, has been demonstrated to induce apoptosis across diverse tumorigenic cell lines, including those of colon, breast, and glioma origin (Kim et al., 2012; Tsai et al., 2012; Yu and Kim, 2011). Moreover, laboratory investigations indicate that wogonin can downregulate the viral E6 and E7 oncogenes as a proximate antiviral action, which subsequently provokes downstream apoptosis in HPV-positive cervical cancer cells, such as CaSki and SiHa (Kim et al., 2013). However, these findings are currently limited to *in vitro* cellular assays. Therefore, further clinical trials and animal studies are warranted to validate these observations.

Bupleuri radix (Ma, 2024) (*Bupleurum chinense* DC. or *Bupleurum scorzonerifolium* Willd.; Apiaceae) yields various bioactive metabolites (Fang et al., 2017; Liu et al., 2020; Bencheraiet et al., 2011; Benedetta et al., 2017), among which saikosaponins are recognized as the principal active metabolites, exhibiting potent anti-inflammatory, antioxidant, and anti-tumor properties (Yu et al., 2020; Tan and Muying, 2021; Cholet et al., 2019; Charu et al., 2013; Shu-Ju et al., 2008; Reneta et al., 2015; Khamjan et al., 2023). Given the established role of ROS in facilitating DNA double-strand breaks and subsequent viral integration, the potent antioxidant capacity of saikosaponin provides a strong mechanistic rationale for its ability to functionally disrupt the persistence loop. Using fluorescence quantitative PCR as a pharmacodynamic readout, treatment with the aqueous extract of Bupleuri radix rendered HPV DNA amplification undetectable after 1 day of exposure, indicating a marked reduction in measurable viral nucleic acid levels. Dose–response assessment further showed a minimum effective concentration of 0.2 g/mL (crude drug equivalent), whereas concentrations ≤0.1 g/mL failed to completely abolish HPV DNA amplification (Li et al., 2005). As an early exploratory investigation, this study focused primarily on demonstrating virological efficacy using tissue homogenates. Whether this specific concentration exerts potential pro-inflammatory or cytotoxic effects on healthy cervical stromal and epithelial cells remains to be evaluated. Therefore, while the loss of HPV DNA amplification serves as a valuable proof-of-concept endpoint, future studies incorporating rigorous local safety and tolerability assays are necessary before such concentrations can be safely translated into clinical topical applications. Through this lens, Bupleuri radix may weaken persistence-supporting DNA states, potentially by mitigating ROS-driven genomic integration events, provided an optimal balance between antiviral efficacy and local tolerability is established.

Collectively, the evidence indicates that suppression of HPV E6/E7 expression represents a central molecular axis through which botanical drugs and their active metabolites exert anti-HPV and anti-cervical carcinogenic effects. Beyond direct inhibition of E6/E7 transcription, multiple metabolites modulate upstream transcriptional regulators, thereby disrupting the transcriptional circuitry that sustains viral oncogene expression. This multi-layered regulatory pattern suggests that botanical drug interventions do not merely silence viral oncogenes, but rather reprogram the viral–host transcriptional network at both transcriptional and epigenetic levels. Such coordinated regulation may underlie their ability to attenuate persistent HPV infection and reverse early malignant transformation, providing a mechanistic basis for their therapeutic potential in HPV-associated diseases.

#### 4.1.2 Inducing viral DNA damage and limiting viral DNA persistence

Reducing viral oncogene output and destabilizing persistence-supporting HPV DNA states can translate into functional restoration of host tumor-suppressive checkpoints and measurable restraint of HPV-driven growth programs. In this context, the most informative downstream readouts are not nonspecific cytotoxic endpoints alone, but checkpoint re-engagement, DNA damage-associated proliferative arrest, and stress-linked apoptotic or autophagic programs that become permissible once HPV-supportive maintenance circuits are weakened. Importantly, these checkpoint restoration and apoptosis-associated programs are explicitly framed as functionally downstream outputs linked to the attenuation of viral maintenance nodes, rather than being treated as independent proof of direct antiviral activity.

In studies summarized above, several phytochemicals that suppress E6/E7 also exhibit checkpoint-linked molecular readouts consistent with re-engagement of p53- and pRb-centered control. For example, matrine decreases E6/E7 transcription and is accompanied by increased expression of cell-cycle inhibitory proteins P21 and P27, and experimental perturbations indicate that P21 contributes to the observed antiproliferative phenotype (Zhang, 2020). In HPV16-infected cellular settings, BJOE is associated with reduced E6/E7 protein levels together with increased Rb protein expression and modulation of mutant p53, linking viral oncogene attenuation to recovery of checkpoint-associated outputs (Guo et al., 2012). In HPV-positive cervical cancer cells, wogonin-induced downregulation of E6/E7 coincides with increased p53 and pRb-related signals, whereas p53 is not altered in HPV-negative cells, supporting HPV-context dependence of this checkpoint restoration pattern (Kim et al., 2013). Berberine likewise promotes restoration of downstream tumor-suppressive restraints in HPV-positive cervical cancer cells. In HPV18-positive HeLa cells, berberine exposure is accompanied by increased p53 expression together with reduced HPV18 E6/E7, and p53 siRNA perturbation experiments further suggest that berberine can at least partially recover p53-linked tumor-suppressive signaling while suppressing HPV18 E7 more prominently than E6. This checkpoint-oriented response is further supported by concomitant reductions in Cyclin, Cdk, and NF-κB expression, consistent with broader re-engagement of cell-cycle restraint and pro-death signaling programs in HPV-positive cervical cancer cells (Saha and Khuda-Bukhsh, 2014).

Consistent with this downstream reprogramming logic, glycyrrhizin also translates suppression of HPV-associated oncogenic signaling into broader loss of proliferative and survival capacity. In HPV16-positive CaSki cells, glycyrrhizin induces G0/G1 arrest, accompanied by reduced Cyclin D1 and CDK4 expression together with increased p21Cip1. In parallel, glycyrrhizin increases intracellular ROS, disrupts mitochondrial membrane potential, activates caspase-8, caspase-9, and caspase-3, upregulates the pro-apoptotic genes Bax and Bad, and downregulates the anti-apoptotic gene Bcl-2. These findings indicate that suppression of HPV E6/E7 and associated host-supportive signaling can be translated into both cell-cycle restraint and apoptotic execution, thereby functionally reinforcing loss of the HPV-dependent transformed state (Ahmad et al., 2022).

Not all relevant downstream restraints, however, require an antecedent E6/E7 readout in the same experimental system. α-Hederin, a pentacyclic triterpenoid saponin isolated from Fructus akebia (Qijuan et al., 2023; Han et al., 2024) (*Akebia trifoliata* Koidz.; Lardizabalaceae), provides a more explicit example of DNA damage-linked checkpoint engagement in cervical cancer cells. In HeLa and SiHa models, α-hederin suppresses proliferation, promotes apoptosis, and induces G2/M arrest, with transcriptomic and mechanistic analyses indicating regulation of cell-cycle, DNA-replication, and P53-related pathways. At the signaling level, α-hederin increases γ-H2AX and engages ATM-CHK1-CDC25B/CDC25C as well as ATM-P53-P21 signaling, thereby suppressing CDK1/Cyclin B activity and enforcing G2/M blockade. This pattern supports the interpretation that, once persistence-supportive malignant programs are destabilized, plant-derived metabolites may convert DNA damage-associated stress into checkpoint-dependent proliferative arrest and apoptotic commitment (Song et al., 2025).

Pachymic acid, the major bioactive of wolfiporia extensa (*Poria cocos* Wolf., Polyporaceae), further broadens the downstream consequence spectrum by promoting autophagic dismantling of cervical cancer cell survival programs. In HeLa and SiHa cells, pachymic acid inhibits proliferation in a concentration-dependent manner and increases autophagosome formation, while Western blot analyses show reduced p-AKT and p-mTOR together with an increased LC3-II/LC3-I ratio. These findings support inhibition of AKT/mTOR signaling with induction of autophagy as a major downstream mechanism. Although pachymic acid has not yet been linked to direct HPV-specific readouts such as E6/E7 suppression, it provides complementary evidence that botanical metabolites may still destabilize persistence-supportive malignant states by forcing tumor cells into stress-associated autophagic programs incompatible with sustained growth (Wu, 2021).

Beyond E6/E7-targeted suppression, interventions that reduce detectable viral DNA burden provide an additional route to weaken the upstream template basis for oncogene expression and thereby favor checkpoint reactivation. In this context, the aqueous extract of Bupleuri radix renders HPV DNA amplification undetectable at an effective concentration, suggesting that depletion of measurable viral nucleic acids may contribute to downstream restoration of tumor suppressor control (Li et al., 2005). Collectively, these functionally oriented readouts connect phytochemical actions at viral maintenance nodes to re-established cell-cycle restraint and apoptosis-associated programs that are directly relevant to limiting lesion persistence and progression.

### 4.2 Reprogramming mucosal immunity and weakening immune evasion nodes

A recurring theme across botanical studies is the capacity to reshape the cervicovaginal immune milieu in ways that may oppose HPV persistence. Rather than acting through indiscriminate immune stimulation, reported botanical effects are more consistently aligned with context-dependent mucosal immune reprogramming, including modulation of cytokine environments linked to antiviral competence, attenuation of intracellular signaling programs that sustain immune escape, and restoration of local conditions permissive for immune-cell recruitment and effector activity. In this section, evidence is summarized using endpoints such as cytokine profiles, phosphorylation and activation states of key signaling pathways, checkpoint-related molecules, and immune-cell recruitment or differentiation readouts.

STAT3 represents one such immune-evasion node, as it links persistent inflammatory input to immunosuppressive and tumor-supportive transcriptional states. In HPV-positive cervical cancer models, the ethanolic mother tincture of berberis aquifolium root reduced total STAT3 and pSTAT3(Y705), particularly in SiHa cells, and this change was accompanied by reduced HPV E6/E7 protein expression (Guo et al., 2011). Although these observations were generated in tumor-cell systems rather than integrated cervicovaginal immune models, they support the concept that botanical interventions may weaken HPV-supportive immune-evasion circuitry by attenuating signaling pathways that help maintain an immunosuppressive state.

A more directly microenvironment-oriented example is provided by borneol (Wu et al., 2001) (*Camphora officinarum* Boerh. ex Fabr.; Lauraceae, or *Dryobalanops aromatica* C.F.Gaertn.; Dipterocarpaceae, or *Blumea balsamifera* (L.) DC.; Asteraceae), a bioactive metabolite traditionally valued for its antibacterial, anti-inflammatory, and analgesic properties (Wu et al., 2020). When combined with other drugs, it is often used as a “guide drug” to enhance their therapeutic efficacy and overcome delivery barriers. Pharmacologically, borneol achieves this “guiding” effect through a dual-layered penetration mechanism. Physically, extensive evidence establishes borneol as a broad-spectrum permeation enhancer across diverse physiological barriers, including mucosal epithelia (Yang et al., 2025; Yanfeng et al., 2025). It transiently and reversibly modulates the expression and structural distribution of critical tight junction proteins, primarily ZO-1 and occludin (Kulkarni et al., 2021; Liang et al., 2023). This modulation reversibly opens paracellular pathways, facilitating the deep tissue penetration of co-administered antiviral metabolites to reach the basal epithelial layers where HPV establishes persistence. Beyond physical drug delivery, borneol exerts a profound “immunological guiding” role. A single-center retrospective cohort analysis (Liu et al., 2025) demonstrated that borneol inhibits the progression of HPV infection by modulating the interplay between innate and adaptive immunity. Specifically, borneol selectively suppresses the secretion of interleukin-6 (IL-6) and interleukin-1β (IL-1β) by macrophages, leading to decreased phosphorylation of STAT3. This reduction inhibits the expression of CD274 (programmed death ligand 1, PD-L1) in infected epithelial cells, limiting their immune evasion capabilities. Additionally, borneol upregulates the expression of tumor necrosis factor-α (TNF-α) and caspase-1 (CASP1) in macrophages, as well as interleukin-12 (IL-12) and interleukin-23 (IL-23) in dendritic cells (DCs), thereby facilitating focal T-cell infiltration, recruitment, and differentiation within the infected microenvironment. The dual capacity of borneol to enhance drug penetration via the transient opening of tight junctions, coupled with its ability to weaken immunological tolerance and facilitate T-cell infiltration, establishes it as a rational adjunct in topical HPV management.

Additional clinical evidence demonstrates that local botanical formulations actively recalibrate broader mucosal immune networks, establishing a cervicovaginal environment distinctly less permissive to HPV persistence. Exemplifying this capacity, the Baofukang suppository, composed primarily of zedoary turmeric oil and borneol, integrates broad antimicrobial, anti-inflammatory, and mucosal-reparative properties with profound local immunomodulation. In patients with cervical HPV infection, Baofukang therapy robustly elevates CD3^+^, CD4^+^, and CD4+/CD8+ ratios while concurrently reducing CD8^+^ populations. These immunological shifts correlate with superior HPV clearance and enhanced symptomatic relief when compared to standard interferon-based local interventions (Song and Bai, 2024). Furthermore, in cases of HPV-complicated cervicitis, the integration of Baofukang with interferon skews the local immune profile toward robust antiviral competence. This combinatorial approach upregulates Th1-cell proportions and T-bet mRNA expression while downregulating Th2-cell populations and GATA-3 mRNA, ultimately driving higher HPV-negative conversion rates and facilitating cytological (LCT) normalization (Zhao et al., 2023). Although these findings are clinically rather than strictly molecularly derived, they substantiate a broader therapeutic paradigm: botanical therapies promote viral clearance not merely via direct antiviral or barrier-restorative effects, but by profoundly rebalancing adaptive immune polarization and reinstating lymphocyte-mediated mucosal immunity.

Importantly, the immunological actions of botanical drugs in cervical HPV settings should not be interpreted as indiscriminate immune enhancement. Rather, their multi-target pharmacology may be better understood as context-dependent mucosal immune reprogramming. In a pre-existing dysbiotic state, where chronic low-grade inflammation, barrier dysfunction, and ineffective antiviral surveillance coexist, excessive non-selective stimulation could theoretically reinforce inflammatory remodeling. However, available botanical immunopharmacology literature suggests that many plant-derived agents can exert bidirectional effects (Liu-hua et al., 2020), enhancing antiviral immune competence while simultaneously restraining maladaptive cytokine overproduction or macrophage hyperactivation depending on the host baseline state (Yin et al., 2019). Accordingly, in HPV persistence, the desired therapeutic direction is not maximal inflammatory activation, but restoration of a balanced mucosal milieu that supports viral clearance without amplifying the chronic inflammatory niche (Bhat et al., 2011).

### 4.3 Resolving chronic inflammation and inflammatory tolerance niches

Beyond direct antiviral and immune-priming effects, plant-derived metabolite shows activity against chronic inflammatory and oxidative programs that help sustain lesion persistence. This inflammation-resolving dimension is particularly relevant in dysbiosis-associated settings, where ineffective antiviral surveillance often coexists with persistent low-grade inflammatory signaling, epithelial barrier disturbance, and microenvironmental remodeling. Under these conditions, the therapeutic objective is not merely to enhance host defense, but also to interrupt the feed-forward molecular programs that convert chronic mucosal perturbation into a self-sustaining, lesion-permissive inflammatory niche (Bhat et al., 2011). This section focuses on inflammation evidence organized around pathway-linked endpoints and lesion-relevant functional outcomes.

Matrine similarly suppresses HPV-positive cervical cancer progression by explicitly targeting the HMGB1/RAGE signaling axis. In HeLa models, matrine treatment attenuated cell viability, invasion, migration, and vascular mimicry *in vitro*, while concurrently inducing apoptosis and downregulating HMGB1 and RAGE protein expression. *In vivo* xenograft studies mirrored these findings, demonstrating diminished tumor growth, reduced CD31-positive angiogenesis, and coordinated suppression of the HMGB1/RAGE pathway. Crucially, ectopic HMGB1 overexpression rescued these malignant phenotypes both *in vitro* and *in vivo*, establishing HMGB1/RAGE signaling as a functional, mechanistic target of matrine rather than a mere downstream correlate (Ran et al., 2026). Given that these readouts predominantly reflect the suppression of inflammation-driven oncogenic signaling, invasive capacity, and angiogenic remodeling, these findings delineate a role for botanical metabolites in dismantling the chronic inflammatory circuits that drive cervical cancer progression, and position the HMGB1/RAGE axis as a pathway-level target.

Glaucocalyxin B, an ent-kaurane diterpenoid isolated from *isodon japonicus* H.Hara, has been reported to exhibit multiple bioactivities, including antitumor and anti-inflammatory effects. Mechanistically, glaucocalyxin B extends the inflammation-resolving framework by acting on the oxidative component of persistence-supportive inflammatory remodeling. In cervical cancer cells, glaucocalyxin B increased intracellular ROS levels in a concentration-dependent manner across C33A, HeLa, and SiHa models, while concurrently suppressing cell viability and clonogenic growth and increasing cleaved PARP and LC3 expression, consistent with induction of apoptosis and autophagy. Importantly, pharmacologic ROS scavenging with N-acetylcysteine attenuated these effects, lowering intracellular ROS and partially restoring proliferative and clonogenic capacity while reducing cleaved PARP and LC3 levels (Liang L., 2022). These findings indicate that the antitumor activity of glaucocalyxin B is not simply accompanied by oxidative stress, but is mechanistically dependent on ROS elevation as an upstream driver of cytotoxic and autophagic responses. In the context of cervical HPV disease, this pattern is relevant because chronic low-grade oxidative remodeling can support maladaptive survival signaling, whereas forcing ROS beyond the tolerable threshold may shift the microenvironment away from persistence-supportive adaptation toward irreversible tumor-cell damage and death. Accordingly, glaucocalyxin B provides mechanistic support for targeting oxidative tolerance as part of a broader strategy to dismantle inflammation-linked niches that facilitate cervical cancer progression.

Clinical investigations of local botanical formulations further indicate that the attenuation of HPV persistence is tightly coupled with measurable reductions in the cervicovaginal inflammatory burden. In high-risk HPV infections, adjunctive Baofukang suppository therapy significantly reduced cervical inflammation scores and downregulated key inflammatory mediators, including TNF-α and TGF-β1. This evidence demonstrates that local botanical interventions actively dismantle persistence-supportive inflammatory networks, rather than merely suppressing isolated virological readouts (Song and Bai, 2024). Corroborating this, parallel clinical observations report diminished IL-1β levels following treatment, linking the resolution of local inflammatory activity directly to improved HPV clearance outcomes (Tian et al., 2024). Although these clinical readouts do not delineate a single canonical anti-inflammatory pathway, they substantiate a critical therapeutic premise: the antiviral efficacy of selected botanical preparations operates in tandem with the quantifiable relief of inflammatory stress within the local cervical milieu.

### 4.4 Restoring cervicovaginal homeostasis

Plant-derived interventions have also been evaluated for their capacity to correct cervicovaginal microenvironmental dysbiosis linked to HPV persistence. Beyond direct antiviral or immune-priming effects, the clinical relevance of these botanical treatments lies in restoring local homeostasis across three interconnected dimensions: correcting microbial imbalance while preserving protective colonization, mitigating the local inflammatory burden, and promoting epithelial repair concomitant with viral clearance. Consequently, this section synthesizes evidence based on clinical endpoints, including microbiota indices, mucosal inflammatory readouts, local symptom and lesion scores, epithelial-barrier integrity, and HPV clearance outcomes.

Sophora flavescens gel, a formulated botanical therapeutic utilized for cervical HPV infection, represents a well-characterized microecology-oriented intervention. Its principal active constituents, the total alkaloids of Sophora flavescens, exhibit a highly targeted dual functionality. Beyond their documented anti-HPV properties, these alkaloids selectively clear common cervicovaginal pathogens, such as *Staphylococcus aureus*, *Escherichia coli*, and *Candida* albicans, without significantly suppressing the protective, resident *Lactobacillus* populations (Zhu and Deng, 2016; Liang, 2018). By alleviating competitive pathogen pressure and ameliorating the local mucosal milieu, this targeted antimicrobial profile actively facilitates the re-establishment of a Lactobacillus-dominant state (Hui-lan WS-b et al., 2020; Li et al., 2025). Clinical evidence reinforces this ecological paradigm. In a prospective randomized controlled trial, the local administration of Sophora flavescens gel over 3 months significantly mitigated nonspecific vaginal mucosal inflammation while preserving the colonization and proliferation of *Lactobacillus* species (Zhao et al., 2016; Liang J. Q., 2022). Specifically, the intervention drove superior HPV clearance rates and shifted the inflammatory (INF) distribution toward a negative or mild status. Crucially, vaginal pH and H2O2 levels remained stable throughout, indicating that the formulation successfully reconditions a dysbiotic microenvironment without disrupting protective colonization or the acidic barrier (Liang, 2018). Collectively, these findings indicate that the clinical benefit of Sophora flavescens gel derives from targeted ecological reconditioning and inflammatory relief, rather than the indiscriminate antimicrobial depletion of the vaginal ecosystem.

The clinical relevance of topical botanical formulations extends well beyond isolated virological and microbiological endpoints. In the management of cervical HPV infection, the prolonged surveillance strategy often termed “watchful waiting” imposes a substantial burden on patients, exposing them to chronic symptoms such as abnormal vaginal discharge, pruritus, mucosal discomfort, and the psychological distress of persistent infection. In this context, localized botanical interventions offer distinct therapeutic advantages by actively restoring the cervicovaginal epithelial landscape while simultaneously resolving the symptomatic burden. Consequently, their clinical utility lies not merely in facilitating viral clearance, but in actively mitigating the physical and psychological toll of persistent infection. Baofukang and Bruceae fructus formulations exemplify this multifaceted clinical benefit. Clinical trials demonstrate that Baofukang-based topical therapy significantly improves vaginal microecological indices, evidenced by lowered vaginal pH and Nugent scores, while concurrently dampening local inflammation and reducing overall symptom severity with high tolerability (Ji, 2020; Huang, 2016). Parallel observations confirm that this treatment drives robust clinical improvements, including the resolution of purulent discharge, abnormal leucorrhea, and cervical erosion. Notably, these microenvironmental repairs coincide with higher HPV-negative conversion rates compared to standard interferon-based local therapies (Tian et al., 2024; Liu, 2024; Qiu et al., 2014; Han, 2014). Further investigations of Baofukang gel reinforce the paradigm that localized botanical therapy tightly couples HPV clearance with mucosal restoration; substantial viral conversion rates consistently parallel the marked regression of cervical columnar ectopy and erosion-like lesions following repeated treatment courses (Zhang et al., 2013; Liu, 2014). Furthermore, in cases of cervicitis complicated by HPV, Baofukang suppositories significantly reduced RLU/CO values and increased viral clearance rates while resolving cervicitis-associated symptoms (Xu, 2021). Clinical data utilizing Bruceae fructus extend this restorative pattern to early precancerous lesions. In patients presenting with CIN I and concurrent high-risk HPV infection, the topical application of Brucea javanica seed infusion for three courses yielded significantly higher rates of both HPV clearance and CIN I regression compared to observation alone. This substantiates the capacity of targeted botanical interventions to drive the normalization of lesion-associated epithelial defects in tandem with a measurable reduction in the viral load. Collectively, these observations dictate that selected topical botanical formulations achieve clinical relevance not simply as passive adjuncts for viral decline, but as active drivers of cervicovaginal homeostasis, symptom resolution, and epithelial recovery throughout the management of persistent HPV infection.

## 5 Limitations

Standard biomedical management for HPV infection varies by disease stage and lesion type, yet no specific antiviral drug is currently available (Wang et al., 2020). Against this background, the present review does not support a uniform conclusion that plant-derived metabolites broadly exert clinically meaningful anti-HPV activity. Instead, the available literature can be organized into three tiers: a narrow group of chemically defined agents with interpretable virological or upstream regulatory effects in HPV16/18-dominant preclinical systems; a larger body of studies showing downstream cytotoxic, checkpoint-restorative, or autophagic consequences in HPV-positive cells; and a clinically more relevant set of local formulation studies, in which improvements in HPV burden, local inflammation, microecological parameters, or lesion-related outcomes have been reported. Framing the literature in this way is central to the present review and explains why our conclusions are more cautious than those of earlier descriptive summaries.

A major limitation is the field’s continued dependence on simplified *in vitro* systems. Although these models are useful for identifying candidate targets and pathways, they do not reproduce the stratified epithelium, mucus barrier, tissue distribution, stromal interactions, local immune surveillance, or microbiota-dependent modulation that shape persistent cervical HPV infection. As a result, observations of viral suppression or immune modulation in isolated cell systems cannot be directly extrapolated to therapeutic efficacy. This limitation is further compounded by the lack of robust high-risk cervical HPV animal models, which continues to restrict assessment of local pharmacokinetics, tissue exposure, immune regulation, and durable viral clearance in physiologically relevant settings. Current organotypic epithelial models are informative for stratification, exposure, and some persistence-related questions, but they still do not fully capture the immune-evasive environment of cervical HPV disease.

Beyond biological modeling, the pharmaceutical interpretability of the literature is also uneven. Some exploratory studies rely on incompletely characterized extracts, whereas others evaluate complex formulations in which activity cannot be attributed to specific metabolites (Hui-lan WS-b et al., 2020; Liang J. Q., 2022; Wang et al., 2021; Tao et al., 2019; Wang et al., 2017; Qin et al., 2024; Luo et al., 2020; Zhang et al., 2022). Even for nominally pure single entities, local residence, mucus penetration, epithelial depth distribution, and pharmacologically meaningful exposure remain largely undefined. This issue is especially important in cervical HPV disease because local delivery is not merely a secondary formulation concern, but a core determinant of translational plausibility. The same problem extends to complex preparations such as Brucea javanica oil emulsion, for which consistent biological output cannot be assumed unless batch release is linked to chemical fingerprinting, quantitative marker profiling, and pharmacology-relevant potency assessment.

Another limitation is evidentiary inflation. Reduction of E6/E7, AP-1-related activity, or detectable HPV DNA burden is more directly relevant to persistence biology than apoptosis, growth inhibition, or cell-cycle arrest in HPV-positive cells. Yet the literature often presents these downstream cellular consequences as though they were equivalent to direct antiviral evidence. In this review, these categories are intentionally separated, because failure to distinguish them risks overstating pharmacological relevance, particularly for metabolites supported mainly by strong *in vitro* phenotypes without corresponding *in vivo*, exposure, or clinical validation. A similar caution applies to microbiota-related findings: although restoration of cervicovaginal homeostasis is clinically important, most current evidence remains associative, and causal proof that these ecological shifts directly mediate sustained HPV clearance is still limited.

Finally, the biological scope of the current evidence remains narrow. Most mechanistic studies are concentrated in HPV16- and HPV18-positive systems, whereas clinical disease is substantially more heterogeneous. Clinical investigations are often short-term, frequently use interferon-based local therapy as an implicit benchmark, and commonly rely on intermediate endpoints such as viral load, RLU/CO, local symptom improvement, or short-interval negative conversion. Although these outcomes are informative, they do not yet establish durable clearance, recurrence reduction, long-term lesion regression, or prevention of progression. Taken together, the literature is best regarded as mechanistically informative but translationally immature: it has generated plausible host-virus-microenvironment hypotheses and several encouraging local clinical signals, but it has not yet established a stable set of pharmacologically validated botanical interventions for persistent cervical HPV infection.

## 6 Future research prospects

Future progress will depend less on expanding the list of reported botanical candidates and more on establishing a stricter translational pathway for those interventions that remain interpretable after critical filtering. The priority is not the continued accumulation of high-concentration cellular activity claims, but the construction of a persistence-relevant evidence ladder linking chemical definition, local delivery, target engagement, tissue-level activity, and clinically meaningful outcomes.

At the preclinical stage, the most useful advances will come from layered validation strategies that better reflect the biology of persistent cervical HPV infection. Organotypic cervical epithelial platforms, cervical tissue explants, *ex vivo* mucus or penetration models, immune co-culture systems, and microbiota-informed settings are each likely to be more informative than any single model alone. Together, these approaches can help determine whether candidate interventions reach biologically relevant epithelial compartments, engage intended molecular targets, and modulate the epithelial-immune-microbial interactions that support persistence. In parallel, studies of extracts, gels, suppositories, oils, and other local preparations should meet a higher standard of chemical transparency, including standardized fingerprinting, marker-metabolite quantification, batch consistency, and explicit reporting of preparation methods.

Local formulation research should be treated as a primary translational arena rather than as a peripheral pharmaceutical detail. Oil-based systems, gels, suppositories, and related cervicovaginal delivery platforms should be optimized for residence time, mucus compatibility, epithelial penetration, release behavior, and tolerability. Before large phase II trials are pursued, early translational studies, including phase 0 or micro-dosing experiments in human cervical tissue, cervical explants, or related *ex vivo* systems, may be especially valuable for confirming local exposure and target engagement of candidate metabolites or formulations. This bench-to-bedside step is particularly important for compounds whose current support still derives mainly from cell-based models.

Future clinical studies should likewise become more mechanistically interpretable. Beyond general HPV positivity or viral load, trials of local botanical preparations should incorporate biomarker frameworks spanning multiple biological layers, including E6/E7-related readouts, local inflammatory cytokines, indicators of mucosal immune tone, and measures of cervicovaginal microecological balance. Where feasible, transcriptomic and microbiomic profiling may help define response heterogeneity and distinguish direct virological effects from broader host-directed mechanisms. Genotype-stratified analyses deserve particular emphasis, especially in populations in which HPV52 and HPV58 are prevalent. Comparator selection should also be handled more critically: interferon-based local therapy should not automatically function as a universal gold standard, and placebo controls, add-on designs, or predefined non-inferiority frameworks may often be more appropriate depending on the formulation and its intended clinical role.

Patient-centered benefit should be incorporated explicitly, but it should not be allowed to substitute for mechanistic proof. Because cervical HPV management often involves prolonged surveillance or watchful waiting, local treatments that reduce discharge, pruritus, mucosal discomfort, or anxiety may still have meaningful clinical value even before durable virological superiority is established. Future trials should therefore include patient-reported outcomes, local tolerability, and symptom burden as formal endpoints, interpreted alongside virological and lesion-related outcomes rather than in place of them. This distinction is important if the field is to avoid repeating one of its current problems: conflating useful local symptomatic relief with definitive evidence of persistence-disrupting efficacy.

The field is therefore likely to advance fastest not by sustaining broad claims that plant-derived compounds are generally anti-HPV, but by narrowing attention to a smaller subset of chemically traceable, locally deliverable, and clinically testable interventions. Standardized local formulations with reproducible composition, acceptable tolerability, and coherent signals in HPV burden, local inflammation, microecology, or lesion-related improvement merit more systematic development than poorly defined single-metabolite claims supported only by high-concentration *in vitro* assays. Through this more selective and mechanistically disciplined strategy, botanical therapies may eventually contribute as credible adjuncts in persistent cervical HPV management, rather than remain a heterogeneous body of promising but weakly translatable observations.
