# Supplementary material for: Cervical human papillomavirus: the therapeutic target of botanical drugs
Source: Front Pharmacol. 2026 Apr 30;17:1822976. doi: 10.3389/fphar.2026.1822976 (PMC13171797; doi:10.3389/fphar.2026.1822976)
Supplement: Supplementary file 1 [file Supplementaryfile1.docx]

Supplementary Material

**Supplementary Table1 Literature search method.**

| **Database** | **Time span** | **Search fields** | **Search strategy** |
| --- | --- | --- | --- |
| **PubMed** | Inception to 28 February 2026 | MeSH Terms, Title/Abstract | **#1** “Human Papillomavirus Infections”[Mesh] OR “human papillomavirus infection”[Title/Abstract] OR “HPV infection”[Title/Abstract] OR “cervical HPV infection”[Title/Abstract] OR “persistent HPV infection”[Title/Abstract] OR “high-risk HPV”[Title/Abstract]  **#2** “Plants, Medicinal”[Mesh] OR “Medicine, Herbal”[Mesh] OR “botanical drug*”[Title/Abstract] OR “herbal medicine”[Title/Abstract] OR “plant-derived metabolite*”[Title/Abstract] OR phytochemical*[Title/Abstract] OR extract*[Title/Abstract] OR formulation*[Title/Abstract] OR berberine[Title/Abstract] OR “Berberis aquifolium”[Title/Abstract] OR glycyrrhizin[Title/Abstract] OR matrine[Title/Abstract] OR “bruceine D”[Title/Abstract] OR “Brucea javanica oil emulsion”[Title/Abstract] OR BJOE[Title/Abstract] OR “Brucea javanica seed infusion”[Title/Abstract] OR “zedoary turmeric oil”[Title/Abstract] OR wogonin[Title/Abstract] OR “Bupleuri radix”[Title/Abstract] OR saikosaponin[Title/Abstract] OR borneol[Title/Abstract] OR “Sophora flavescens gel”[Title/Abstract] OR “Baofukang suppository”[Title/Abstract] OR “Baofukang gel”[Title/Abstract] OR “alpha-hederin”[Title/Abstract] OR “α-hederin”[Title/Abstract] OR “pachymic acid”[Title/Abstract] OR “glaucocalyxin B”[Title/Abstract]  **Final search:** **#1 AND #2** |
| **Web of Science** | Inception to 28 February 2026 | Topic (TS = title, abstract, author keywords, Keywords Plus) | **TS**=((“human papillomavirus infection” OR “HPV infection” OR “cervical HPV infection” OR “persistent HPV infection” OR “high-risk HPV”))  **AND**  **TS**= ((“botanical drug” OR “herbal medicine” OR “plant-derived metabolite” OR phytochemical* OR extract* OR formulation* OR berberine OR “Berberis aquifolium” OR glycyrrhizin OR matrine OR “bruceine D” OR “Brucea javanica oil emulsion” OR BJOE OR “Brucea javanica seed infusion” OR “zedoary turmeric oil” OR wogonin OR “Bupleuri radix” OR saikosaponin OR borneol OR “Sophora flavescens gel” OR “Baofukang suppository” OR “Baofukang gel” OR “alpha-hederin” OR “α-hederin” OR “pachymic acid” OR “glaucocalyxin B”)) |
| **CNKI** | Inception to 28 February 2026 | Subject / Title / Keyword / Abstract | **SU/TI/AB/KY =** (宫颈HPV感染 OR 人乳头瘤病毒感染 OR 宫颈人乳头瘤病毒感染 OR 持续性HPV感染 OR 高危型HPV OR 高危型人乳头瘤病毒) **AND** (植物药 OR 中药 OR 中草药 OR 草药 OR 植物来源活性成分 OR 植物代谢产物 OR 提取物 OR 制剂 OR 小檗碱OR 甘草酸 OR 苦参碱 OR 鸦胆子OR 莪术OR 汉黄芩素 OR 柴胡提取物 OR 柴胡皂苷 OR 冰片 OR 苦参凝胶 OR 保妇康栓 OR 保妇康凝胶OR 茯苓酸 OR 蓝萼乙素) |

**Note:** (1) *The Pharmacopoeia of the People’s Republic of China* (Part I, 2025 edition) was consulted for official botanical identification and quality-control information. Reference lists of relevant reviews and included studies were also screened to identify additional eligible records. (2) Mechanism-oriented terms (e.g., oxidative stress, inflammaging) were used in supplementary searches to facilitate retrieval of pathway-focused studies.
