# Supplementary material for: Cervical human papillomavirus: the therapeutic target of botanical drugs
Source: Front Pharmacol. 2026 Apr 30;17:1822976. doi: 10.3389/fphar.2026.1822976 (PMC13171797; doi:10.3389/fphar.2026.1822976)
Supplement: Supplementary file 2 [file Supplementaryfile2.docx]

Supplementary Material

**Supplementary Table 2 Simplified reporting-based checklist of key items sensitive to bias.**

| **Study** | **Evidence type** | **Control groups reported** | **Vehicle and vehicle control reported** | **Dose or final concentration reported** | **Route, frequency, duration, and timing reported** | **Intervention identifiers reported** | **Overall notes**  **(optional)** |
| --- | --- | --- | --- | --- | --- | --- | --- |
| Mahata et al., 2011 | *In vitro* (SiHa/HeLa, C33a, normal peripheral blood lymphocytes) | R | R | R | R | R | - |
| Saha et al., 2014 | *In vitro* (HeLa/C33a/WRL-68) | R | R | R | R | R | - |
| Singh et al., 2021 | *In vitro* (SiHa/HeLa/C33a) | R | R | R | R | R | Commercial mother tincture and ethanol vehicle are clearly reported, but individual constituent standardization is incomplete. |
| Ahmad et al., 2022 | *In vitro* (CaSki) | R | P | R | R | R | Stock was prepared in DMSO, but an explicit matched DMSO-only vehicle control is not clearly described. |
| Zhang et al., 2020 | *In vitro* (SiHa/CaSki) | R | P | R | R | R | Vehicle matching for the complete DMEM medium preparation is not explicitly described as having a matched solvent control beyond "blank". |
| Pan et al., 2015 | *In vitro* (Ect1/E6E7/CaSki) | R | P | R | R | R | Stock was prepared in DMSO, but the control is described as "untreated" without explicit DMSO vehicle matching. |
| Sun et al., 2017 | *In vitro* (Ect1/E6E7/CaSki) | R | P | R | R | R | Stock was prepared in DMSO, but a DMSO-matched vehicle control is not clearly reported. |
| Hu et al., 2013 | *In vitro* (Ect1/E6E7/CaSki) | R | NR | R | R | R | A matched vehicle control for the commercial emulsion injection is not explicitly detailed. |
| Zhang et al., 2014 | *In vitro* (SiHa/CaSki/H8) | R | NR | R | R | R | Solvent or vehicle for the volatile oil preparation and a matched vehicle control are not reported. |
| Kim et al., 2013 | *In vitro* (CaSki/SiHa/C33A) | R | NR | R | R | R | - |
| Li et al., 2005 | *In vitro* (HPV DNA assay) | R | R | R | R | P | Purity, and physicochemical characterization of the aqueous extract are not reported. |
| Song et al., 2025 | *In vitro* (SiHa/HeLa);  *In vivo* (HeLa xenograft/nude mice) | R | R | R | R | R | - |
| Wu, 2021 | *In vitro* (HeLa/SiHa/LO2) | R | P | R | R | P | Stock was prepared in DMSO, but a matched vehicle control is not explicitly described; purity characterization is incomplete. |
| Liu et al., 2025 | *In vitro* (Macrophages, DCs, TC-1, splenic lymphocytes)  *In vivo* (C57BL/6 mice) | R | R | R | R | R | - |
| Tian et al., 2024 | *Clinical* (patients with cervical HPV infection) | R | P | R | R | R | No matched placebo suppository is used. |
| Zhao et al., 2023 | *Clinical* (patients with cervical HPV infection/cervicitis) | R | P | R | R | R | No matched placebo suppository is used. |
| Ran et al., 2026 | *In vitro* (HeLa)  *In vivo* (HeLa xenograft/nude mice) | R | P | R | R | R | - |
| Liang, 2022 | *In vitro* (C33A/HeLa/SiHa) | R | P | R | R | R | Stock was prepared in DMSO, but an explicit matched DMSO vehicle control is not clearly detailed. |
| Song et al., 2024 | *Clinical* (patients with high-risk HPV infection) | R | P | R | R | R | No matched placebo suppository is used. |
| Zhao et al., 2016 | *Clinical* (Patients with cervical HPV infection) | R | R | R | R | R | - |
| Liang, 2018 | *Clinical* (Patients with cervical HPV infection) | R | P | R | R | NR | No matched placebo vehicle is used; control is follow-up only. |
| Huang, 2016 | *Clinical* (patients with CINⅠ and high-risk HPV infection) | R | P | R | R | P | No placebo control is used; the vehicle is described by preparation procedure, but no formal physicochemical standardization or batch identifier is reported. |
| Han, 2014 | *Clinical* (patients with high-risk cervical HPV infection) | R | P | R | R | R | No matched placebo gel is used; comparator is no-treatment follow-up. |
| Zhang et al., 2013 | *Clinical* (patients with cervical HPV infection) | R | P | R | R | R | No matched placebo gel is used; comparator is no-treatment control. |
| Liu, 2014 | *Clinical* (patients with high-risk cervical HPV infection) | R | P | R | R | R | No matched placebo gel is used; comparator is no-treatment control. |
| Liu, 2024 | *Clinical* (Patients with cervical HPV infection) | R | P | R | R | NR | No matched placebo suppository is used. |

**Note:** This table summarizes reporting completeness for key bias sensitive items and is not intended as a subjective risk rating. Codes are defined as follows: **R**, reported with sufficient detail to allow verification or reproducibility; **P**, partially reported, with one or more key details missing; **NR**, not reported or not determinable from the article text and supplementary materials. In the vehicle domain, “vehicle” includes the solvent where applicable. “Intervention identifiers” refer to the intervention name and form, and source or supplier information when available.
